# Spatiotemporal Differentiation of Alpine Butterfly *Parnassius glacialis* (Papilionidae: Parnassiinae) in China: Evidence from Mitochondrial DNA and Nuclear Single Nucleotide Polymorphisms

**DOI:** 10.3390/genes11020188

**Published:** 2020-02-11

**Authors:** Ruisong Tao, Chang Xu, Yunliang Wang, Xiaoyan Sun, Chunxiang Li, Junye Ma, Jiasheng Hao, Qun Yang

**Affiliations:** 1 College of Life Sciences, Anhui Normal University, Wuhu 241000, China; trs.cm@163.com (R.T.); chxu199426@126.com (C.X.); wyl1119@163.com (Y.W.); 2 College of Life Sciences, Hefei Normal University, Hefei 230000, China; 3 SKLPS and Center for Excellence in Life and Paleoenvironment, Nanjing Institute of Geology and Palaeontology, Chinese Academy of Sciences, Nanjing 210008, China; xysun@nigpas.ac.cn (X.S.); cxli@nigpas.ac.cn (C.L.); jyma@nigpas.ac.cn (J.M.); 4 College of Earth and Planetary Sciences, University of Chinese Academy of Sciences, Beijing 100049, China

**Keywords:** *Parnassius*, *Parnassius glacialis*, phylogeny, phylogeography, divergence time estimation, quaternary climatic oscillations

## Abstract

The Apollo butterfly, *Parnassius glacialis*, is one of the most charming members of its genus and includes two subspecies locally distributed in montane areas of south-central China and Japan. In this study, we investigated the genetic structure and demographic history of *P. glacialis* by analyzing partial sequences of four mitochondrial genes and nuclear single nucleotide polymorphisms (SNPs) via genotyping-by-sequencing (GBS) of samples from nearly the entire known distributional range in China. The mitochondrial DNA (mtDNA) data demonstrated that a total of 39 haplotypes were present, and the species was estimated to have diverged about 0.95 million years ago during the middle Pleistocene transition into two main clades that likely formed during the Kunlun-Huanghe tectonic movement. The two clades then dispersed independently in distinct geographic areas alongside the mountainous routes in central and southern China, most likely driven by the Pleistocene glacial-interglacial cycles. Nuclear SNP analysis was generally congruent with mtDNA results at the individual level. A minor incongruence of genetic structures that was detected between mtDNA and nuclear SNP data from the Laojunshan and Tiantangzhai populations was likely due to secondary contact and male-biased dispersal. Our work demonstrates that complicated dispersal-vicariance evolutionary processes likely led to the current geographic distribution of *P. glacialis* in China, particularly the uplift of the Qinghai-Tibet Plateau and related climatic oscillations during the Quaternary period.

## 1. Introduction

The geographic distribution and biodiversity of organisms is strongly influenced by geological events and associated climate variations. Geological events give rise to habitat fragmentation and subsequent spatial restriction of gene flow, a contributing factor in genetic diversification and speciation [1,2,3]. Climate variations are largely responsible for population isolation leading to population divergence [4,5]. For example, glacial-interglacial cycles in the Quaternary period caused periodic expansion and contraction of population distribution area and size; temperate plants and animals retreated to the south during glacial periods and dispersed northward during interglacial times [4,6,7,8,9,10,11]. Major geological and paleoclimatic events in China during the Quaternary period, including the uplift of the Qinghai-Tibet Plateau (QTP) and the formation of glacial landforms in mountain ranges, had profound influences on environmental changes and the evolution of organisms. The uplift of QTP is thought to be responsible for the formation and diversification of endemic species. Compared to climate changes in North America and Europe during the Quaternary period, the uplift of QTP and associated paleoclimatic events resulted in a less severe and extensive glacial advance in East Asia and a warmer climate in southern China [12,13,14,15,16,17].

*Parnassius*, a butterfly genus in the family Papilionidae, is distributed in high-altitude mountains across Asia, Europe, and North America [18]. Because *Parnassius* flies slowly and does not migrate long distances, it is isolated in small local populations in various ecological areas. Additionally, these organisms display a unique reproductive strategy in that the males have special accessory glands that produce a mating plug that seals the female genitalia after mating. This is believed to ensure the success of the male by preventing other males from mating, and therefore avoiding sperm competition [19]. *Parnassius* has diversified into about 60 species, mostly occurring in the Himalayan-Tibetan mountains [20,21]. In China, only one species, *Parnassius glacialis,* has dispersed into areas south of the Yangtze River, inhabiting lower-altitude mountains (~200 to 2000 m). This species remarkably resembles its closest relative *Parnassius stubbendorfii*, which is distributed in northern areas of Asia. Both are commonly characterized by ice-like, clean, and hairy bodies and use *Corydalis yanhusuo* and *C. caudata* as their host plants, respectively [22,23]. However, little is currently known about their spatiotemporal origin and divergence. 

Animal mitochondrial DNA (mtDNA) sequences are maternally inherited, lack recombination, and evolve rapidly as compared with nuclear DNA fragments [24,25]. Mitochondial DNA has been widely used to study molecular evolution, phylogenetics, and population genetics in animals for several decades [26,27]. In recent years, genotyping-by-sequencing (GBS) has allowed for the development of reduced representation libraries of genome-wide single nucleotide polymorphisms (SNPs) from high-throughput sequencing data. It has also been used to estimate the phylogenetic relationship and genetic diversity of animal species [28,29,30,31]. In this study, we attempted to reconstruct the phylogeny, genetic structure, and demographic history of *P. glacialis*, aiming to clarify the spatiotemporal pattern of this species by using mtDNA and nuclear SNP data combined with evidence of paleogeographic and paleoenvironmental evolution in the region.

## 2. Materials and Methods

### 2.1. Specimen Collection

**Figure 1:**
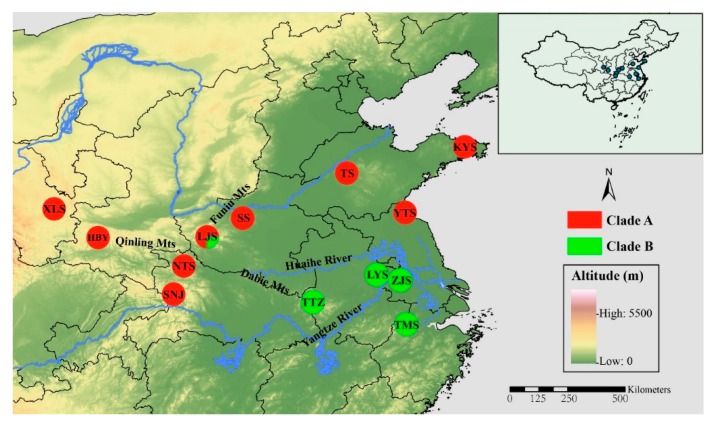
Geographic distribution of the 13 sampled populations of *Parnassius glacialis*. Populations are represented by pie charts with slice size proportional to the frequency of mitochondrial DNA (mtDNA) haplotype clades (see text for explanation). Pie chart size corresponds to the clades in Table 1.

Adult *P. glacialis* individuals (*n* = 368) were collected from 13 localities in central and southern China, covering nearly all known distributional ranges in China. Sampling strategies varied for mtDNA and SNP analyses. All individuals were examined for mtDNA analysis. For SNP analysis, 129 samples from all 13 localities were sequenced (*n* = 9 to 10 samples per locality). The sample size and geographical coordinates for each population are presented in Table 1, and localities are shown in Figure 1. Adult individuals from the closely related species *P. stubbendorfii, P. cephalus, P. imperator*, *P. jacquemontii*, *P. apollonius*, *P. szechenyii*, *P. orleans*, *P. andreji*, *P. choui*, *P. epaphus*, *P. acco*, *P. apollo*, *P. nomion*, *P. actius*, and *P. simo* were also collected from QTP and other areas of China (*n* = 68, Table S1). After sample collection and species identification, fresh samples were promptly fixed in 100% ethanol and preserved at −20 °C for subsequent experiments. 

### 2.2. DNA Extraction, PCR Amplification, and Sequencing

Total DNA was extracted from adult leg muscles with a DNA extraction kit (Sangon Biotech, Shanghai, China) following the manufacturer’s instructions. Amplification of four mtDNA segments (*ND1*, *ND5*, *COI*, and *Cytb* genes) was conducted using primers described in previous reports [32,33,34]. All primers were synthesized by Sangon Biotechnology Co. Ltd. (Shanghai, China). PCR was performed using the following cycling parameters: Initial denaturation for 2 min at 94 °C, 35 cycles of 1 min at 94 °C, 1 min at 46 to 57 °C, and 1 min at 72 °C, followed by final extension for 10 min at 72 °C. Amplified fragments were purified using a DNA Purification Kit (Tiangen Biotech, Beijing, China) and sequenced with an ABI 3730 × l DNA analyzer (Anhui General Biotechnology Co., Ltd., Chuzhou, China) using their corresponding primers and ABI BigDye Terminator v3.1 (Applied Biosystems, Foster City, CA, USA). Raw sequences of the four gene fragments were visually inspected in ClustalX 2.1 software [35]. Subsequently, sequence data were aligned using MUSCLE in MEGA6 software [36] according to amino acid sequence similarity and concatenated into one dataset by DAMBE v7.0.35 software [37].

Genotyping-by-sequencing analysis was conducted according to a slightly modified protocol reported previously [28,38,39]. Whole genome DNA (100 ng/sample) was digested with *Mse*I and *Eco*RI (New England Biolabs, Beverly, MA, USA). The adapter, which contained a barcode sequence, was ligated to the *Mse*I restriction site for each individual sample and another adapter was ligated to the *EcoR*I restriction site of genomic DNA for all samples. After ligation, individual reactions were pooled and purified with Agencourt AMPure XP beads (Beckman Coulter, Miami, FL, USA). PCR amplification was performed using Illumina Primers (Illumina, San Diego, CA, USA) and the following parameters: Temperature cycling at 72 °C (5 min), 98 °C (30 s), followed by 16 cycles of 98 °C (30 s), 65 °C (30 s), and 72 °C (20 s), and a final extension step. PCR products were purified using Agencourt AMPure XP beads (Beckman) and pooled, then, separated on 2% agarose gel for detection. The PCR fragments (375 to 400 bp, including indexes and adaptors) were isolated using a Gel Extraction Kit (Qiagen, Hilden, Germany) and purified using Agencourt AMPure XP beads. Paired-end sequencing (minimum depth coverage = 10) was performed on selected tags using an Illumina HiSeq 2000 (Illumina). Sample sequences were sorted according to the barcodes. To ensure the reliability of reads for population genetics analysis, raw reads were processed through a series of quality control procedures [40]. Both GBS library preparation and Illumina sequencing were performed by NovoGene Co. Ltd. (Beijing, China).

### 2.3. Mitochondrial Data Analysis

In this study, 368 individuals of *P. glacialis*, 68 adult individuals from closely related species, and *Hypermnestra helios (LS975127)* served as the ingroup, while the three Parnassiinae species *Luehdorfia taibai* (NC023938), *L. chinensis* (KU360130), and *Sericinus montelus* (HQ259122) served as the outgroups. Bayesian inference (BI) analysis was performed to construct the phylogeny based on concatenated datasets of four mitochondrial genes (2994 bp in total) using MrBayes 3.2 [41]. On the basis of Akaike information criterion (AIC), the GTR+I+G model was selected via jModelTest 2.1.6 [42]. Ten million generations, with sampling at every 1000th generation, were processed and two separate runs consisting of four Markov chains (one cold and three heated) were conducted simultaneously. Convergence of the runs was assessed using the potential scale reduction factor (PSRF = 1.0) and average standard deviation of split frequencies (<0.01), with the first 2500 (25%) generations discarded as burn-in samples. Bayesian posterior probabilities were estimated from the posterior distribution of remaining trees [43]. A haplotype network was implemented using a median-joining network algorithm in NETWORK v5.0.1 (http://www.fluxus-engineering.com), with network loops resolved via coalescence theory-based criteria. 

The number of haplotypes (*h*), haplotype diversity (*H_d_*), and indices of nucleotide diversity (*π*) were calculated for each population using DnaSP 5.10 [44]. Nei’s pairwise genetic distance matrix among populations, analysis of molecular variance (AMOVA), and Mantel tests (estimation of relative contributions between genetic distance and geographical distance) were performed using GenAlEx v6.1 [45]. 

On the basis of the mtDNA data, a time-calibrated phylogeny of the *Parnassius* species was estimated using BEAST v1.83 [46]. For molecular dating analysis, the clock model was set as relaxed, uncorrelated lognormal with the tree prior set as the birth-death process. The MCMC chain was run for 10 million generations to achieve convergence and sampled every 1000 generations. Convergence was assessed in the systematic sample after a 10% burn-in using Tracer v1.7 [47]. A maximum clade credibility tree was generated by the Tree Annotator program within BEAST. The final chronogram and node ages were visualized in FigTree v1.4.3 [48].

Swallowtail butterfly fossils (family Papilionidae) are scarce, with the oldest papilionid fossils reported from the Lutetian Stage (41.2 to 47.8 Ma, mid-Eocene) of the Green River Formation (USA, Colorado), and two unambiguous fossils of Parnassiinae, *Thaites ruminiana* from the Chattian Stage (23.03 to 28.1 Ma, late Oligocene), and *Doritites bosniaskii* from the Messinian Stage (5.33 to 7.25 Ma, late Miocene) [49,50,51]. In the analysis, the minimum age for the crown of Parnassiinae was constrained to be 23.03 Ma based on the closely related fossil *Thaites ruminiana.* The initial split of genus *Parnassius* was constrained to be 13 to 22 Ma [52], and the last common ancestor of *Luehdorfia* was set to be 9.6 ± 1.2 Ma [53].

Ancestral area reconstructions were inferred using RASP 3.2 [54] and the statistical dispersal-vicariance method (S-DIVA) [55]. To reconstruct ancestral areas on the basis of topography, the distribution of *Parnassius* in China was divided into five regions as follows: Qinling Mountains-Huaihe River region (A), area south of the Huaihe River (B), northeast China (C), Xinjiang (D), and the QTP (E) (C and D regional data not included for this study, only analyzed for comparison). Time trees used in this analysis were generated by BEAST v1.83. The ancestral area was reconstructed by obtaining the marginal probabilities of alternative ancestral distributions through statistical dispersal-vicariance analysis, and frequencies of an ancestral range at a node were averaged over all trees.

Neutrality tests were performed in DnaSP 5.10. Tajima’s *D* [56] and Fu’s *Fs* [57] statistics were applied to detect significant deviation from the assumption of neutrality with 10,000 bootstrap replicates. Demographic change was estimated using mismatch distributions based on concatenated sequences in Arlequin v.3.5.158 [58]. Validity of the expansion model was assessed with the Harpending raggedness index (*r*) and the sum of squared deviations (*SSD*) between expected and observed values [58,59]. When evidence of population expansion was found, two Bayesian coalescent analysis models (Bayesian skyline plot and constant-size model) were applied using BEAST v1.83 in order to determine a suitable substitution rate of *P. glacialis*. Relative fit of the two models was determined by calculating approximated Bayes factors via estimation of marginal likelihoods, using the algorithm implemented in Tracer v1.6 [8]. Expansion time (t, number of generations) was estimated with the formula *t* = *τ*/2*u* [60], where *τ* is the parameter of sudden expansion and *u* is the mutation rate per generation for the entire sequence under study (calculated using the equation *u* = 2*µk*, where *µ* is the substitution rate per site per generation and *k* is the sequence length per haplotype). Considering *P. glacialis* are univoltine with non-overlapping generations, generation time was set to one year. Furthermore, Bayesian skyline plot (BSP) analysis was used to estimate the change in population size with time in BEAST v1.83. The piecewise-constant skyline model was selected, and a relaxed uncorrelated lognormal molecular clock was used. The mutation rate (substitution per site per MY) was selected as described above.

### 2.4. Nuclear Genotyping-by-Sequencing (GBS) Analysis

Determined sequences without barcodes, restriction site remnants, and “N’s” were discarded to filter out false positive SNPs. Filtered raw sequences were assembled with a short-read assembling program SOAPdenovo [61]. After quality filtering and sequence assembly, SNP calling was performed by SAMtools 1.3 [62], and SNPs with more than 50% missing data or minor allele frequency (MAF) below 0.01 were removed.

Evolutionary relationships and the population structure were characterized using three complementary approaches: (1) individual-based neighbor joining (NJ) trees were constructed based on *p*-distance using TreeBeST v1.9.2 [63], (2) principal components analysis (PCA) was performed using GCTA [64], and (3) population genetic structure of *P. glacialis* individuals was determined by ADMIXTURE v1.3.0 [65]. 

## 3. Results

### 3.1. Mitochondrial DNA

The alignments for mitochondrial *ND1*, *ND5*, *COI*, and *Cytb* segments were 936, 750, 648, and 663 bp, respectively. The aligned concatenated nucleotide dataset contained a total of 2994 bp, and no indels or stop codons. All gene sequences were deposited into GenBank acquiring the accession numbers as MH518317 to MH520060. Polymorphic sites were observed with frequencies of 7, 2, 9, and 14 for *ND1*, *ND5*, *Cytb*, and *COI*, respectively, defining all 39 haplotypes for 368 individuals of *P. glacialis* from 13 localities (Figure 1 and Table 1). The percentage of sequence divergence among 39 haplotypes ranged from 0.03% to 0.54%, with an average of 0.22%.

**Figure 2:**
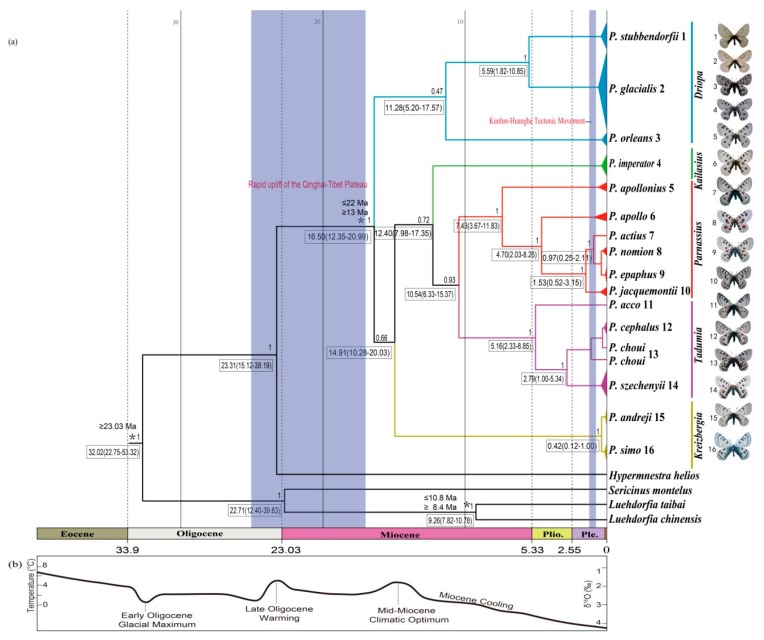
Estimated time tree of *Parnassius* based on mitochondrial sequences in relation to geological and climatic events from the Oligocene epoch to the present. (**a**) Divergence time estimated from BEAST analysis, with relevant geological events (blue shades), 95% highest posterior density (HPD) intervals (boxes) and calibration points (*). Numbers above branches represent Bayesian posterior probability (PP); (**b**) global climate curve, from Zachos et al. [66].

**Figure 3:**
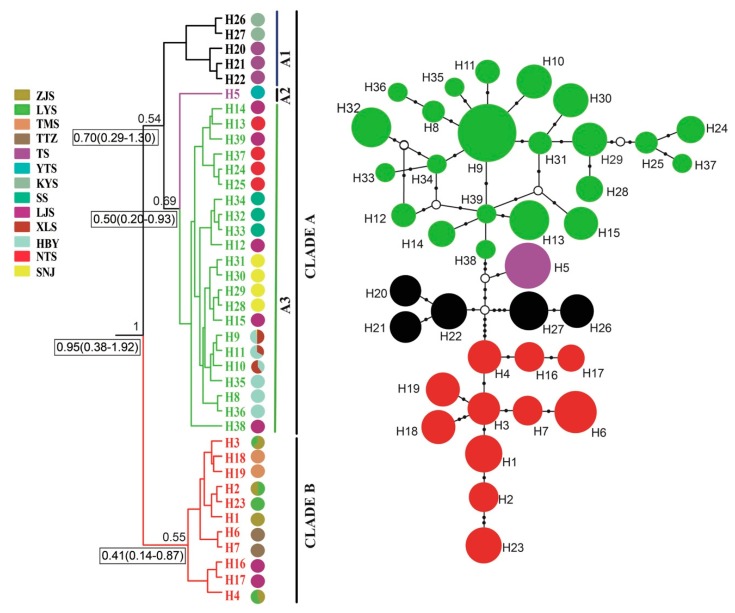
Bayesian phylogenetic tree (left) and median-joining network (right) based on mtDNA haplotypes of *P. glacialis*. Haplotype frequency denoted by circle size, black dots represent corresponding mutation steps, and open circles indicate undetected haplotypes.

A phylogenetic tree inferred using Bayesian inference (BI) indicated that the 16 *Parnassius* species in this study could be assigned to the five major subgenera (*Driopa*, *Kailasius*, *Tadumia*, *Parnassius*, and *Kreizbergia*). *P. glacialis* was sister to *P. stubbendorfii*, which constituted a clade sister to the grouping of all other *Parnassius* species (Figure 2). In the Bayesian tree (Figure 3), the 39 *P. glacialis* haplotypes were distinctly split into two monophyletic haplogroups, Clade A and Clade B. Clade A included 28 haplotypes that were widely distributed in the Qinling Mountains (XLS, HBY, NTS, SNJ, SS, and LJS-partial) and the region north of the Huaihe River (TS, KYS, and YTS) (Figure 1). Clade A was further divided into three subclades of specific geographic areas, i.e., A1, A2, and A3. Subclade A1 (TS and KYS) and Subclade A2 (YTS) were from the Shandong Peninsula and Northern Jiangsu Province, respectively, and Subclade A3 was mostly from the Qinling Mountains and adjacent regions. Clade B consisted of 11 haplotypes from areas south of the Huaihe River (TTZ, LYS, ZJS, and TMS), and a subset of the population from LJS. The reconstructed haplotype median-joining network showed that the distribution of haplotypes was consistent with the phylogenetic tree (Figure 3). 

Haplotype, haplotype diversity (*H_d_*), and nucleotide diversity (*π*) for all populations of *P. glacialis* are summarized in Table 1. Thirty-two of the 39 haplotypes (82.05%) were private haplotypes, each found in only one population. The remaining seven haplotypes (17.95%) were shared among two or more populations. The most abundant haplotype H9, shared by 38 individuals, was distributed in three populations (HBY, XLS, and SNJ). Each population had at least two haplotypes, except for the YTS population, which had only one haplotype (H5) with the lowest *H_d_* (0). The highest haploid diversity (*H_d_* = 0.754) was detected in the LJS population, with a nucleotide diversity (*π*) of 0.00188. 

**Figure 4:**
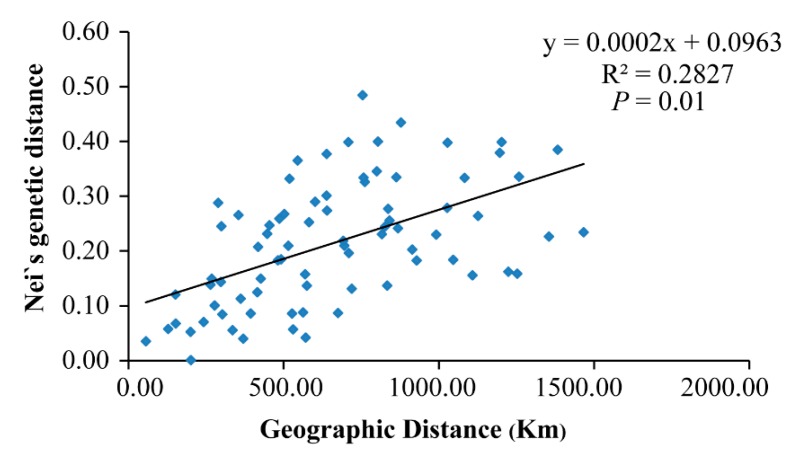
Scatter plot of Nei’s genetic distance vs. geographical distance (based on longitude and latitude coordinates) for *P. glacialis* populations (Mantel test).

Nei’s genetic distance between populations ranged from 0.001 (between HBY and XLS) to 0.484 (between LYS and SNJ) (Table 2). The mean distance between Clade A and Clade B was ~0.21, while mean distances among populations in Clades A and B were ~0.15 and ~0.08, respectively. The estimated mean gene flow, Nm, among populations was 0.136. A positive correlation was found between genetic distance and geographic distance (Mantel test, *p* = 0.01, R^2^ = 0.28) (Figure 4), which indicates that the genetic differentiation among populations of *P. glacialis* was at least somewhat associated with isolation by distance. AMOVA analysis indicated that nearly half of the genetic variation occurred between the two clades (46.37%), while 37.68% of variation occurred among populations and 15.95% occurred within populations (Table 3).

Divergence times between species/major clades in this study were estimated as shown in Figure 2. The most recent common ancestor (MRCA) of *Parnassius* was dated at ~16.50 Ma with a 95% highest posterior density (HPD) at 12.35 to 20.99 Ma (early Miocene); the split of *P. glacialis* and *P. stubbendorfii* was estimated at ~5.59 Ma (95% HPD, 1.82 to 10.85 Ma, late Miocene). The divergence of Clades A and B of *P. glacialis* was ~0.95 Ma (95% HPD, 0.38 to 1.92 Ma, early-middle Pleistocene). 

**Figure 5:**
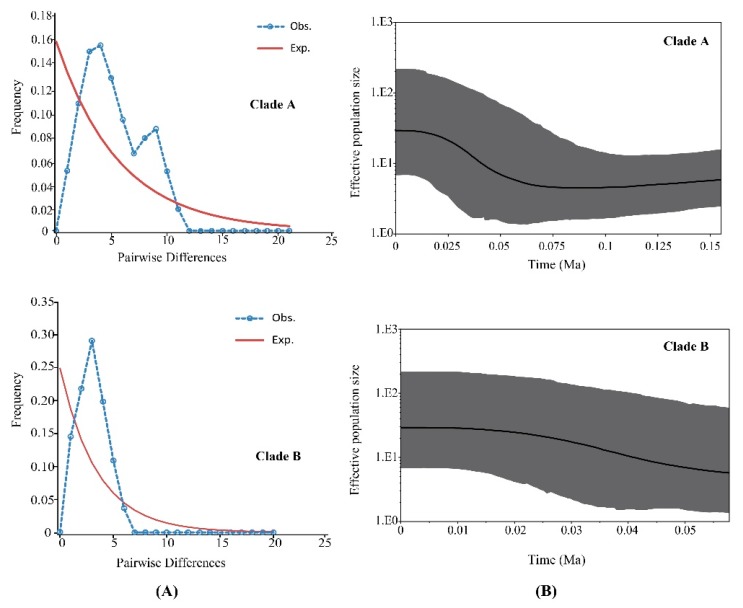
Demographic expansion models for two major clades of *P. glacialis*. (**A**) Mismatch distribution analyses for Clades A and B. The blue dotted and brown solid lines represent the observed and expected mismatch distributions of a stationary population, respectively; (**B**) Bayesian skyline plots for Clades A and B. The *X*-axis represents time (Ma) and the *Y*-axis is Ne × μ (effective population size × mutation rate per site per generation). Medians are shown as solid lines, and gray areas represent 95% HPD limits.

Clades A and B were analyzed by mismatch distribution analysis and neutrality tests to discover historical demographic changes in *P. glacialis* (Figure 5A and Table 4). Clade A exhibited a smooth bimodal frequency distribution, and *SSD* and *r*-values from the expected curve were not significant. However, the Clade A neutrality test indicated that Fu’s *Fs* was non-significant and negative, indicating a disagreement in outcomes for this clade. For Clade B, the mismatch distribution was unimodal and both *SSD* and *r* index tests failed to reject the hypothesis of a sudden expansion model. A neutrality test of Clade B resulted in significant negative values for Tajima’s *D* and Fu’s *Fs* statistics, indicating that Clade B experienced recent demographic expansion.

Bayesian skyline plot analyses based on coalescent theory revealed detailed demographic histories for both clades. The population size of Clade A was relatively stable for a long period, followed by expansion at ~56 Ka (Figure 5B). The demographic expansion of Clade B began approximately at 45 Ka.

**Figure 6:**
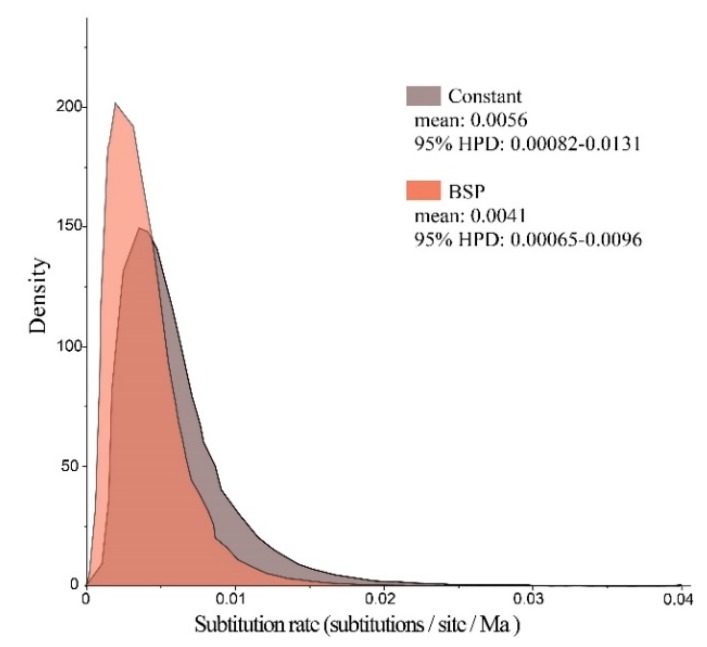
Marginal posterior probability distributions for substitution rate of *P. glacialis* mtDNA modeled by constant-size and Bayesian skyline plot (BSP) methods. Molecular dating was calibrated by assuming a time to Most Recent Common Ancestry of 0.95 Ma for *P. glacialis*.

Bayes factors were calculated for two coalescent models (log_10_ BF = 0.56) to develop a constant-size coalescent model. The molecular evolutionary rate was modeled as a bell-shaped marginal posterior probability distribution with a mean value of 0.56% substitutions per site per million years (95% HPD, 0.082% to 1.31%, Figure 6). On the basis of the observed values for age expansion (τ = 3.018, Table 4) and substitution rate (of 0.56% per site per Ma), the demographic expansion time of Clade B was estimated to be approximately 44.8 Ka.

### 3.2. Nuclear Single Nucleotide Polymorphisms (SNPs)

The GBS library, sequenced using Illumina technology, yielded a total of 60.28 Gbp of sequence data averaging 478.45 Mbp per sample. On average, the GBS library produced 1,658,046 raw de-multiplexed reads. The sequence data was high quality (Q20 ≥ 96.98% and Q30 ≥ 85%), GC percentage ranged from 39.17 to 42.53, and mean GC percentage was 40.94. The SNP calling pipeline identified 1,040,945 SNPs. After quality control, 166,546 SNPs were kept for subsequent analysis. The raw sequencing reads were deposited in the BioProject, accession number: PRJNA603210, and BioSample, under the following accession number: SAMN13924708-SAMN13924836.

**Figure 7:**
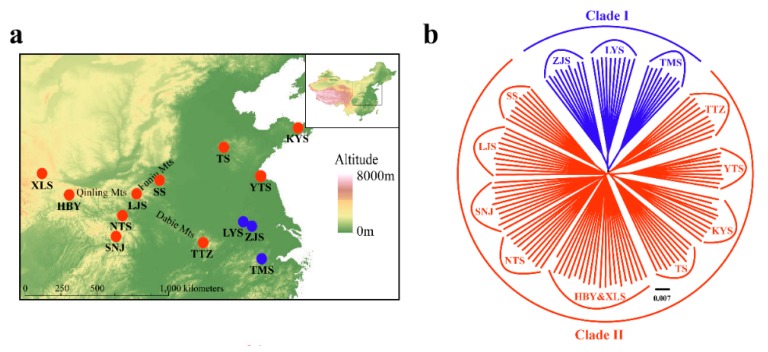

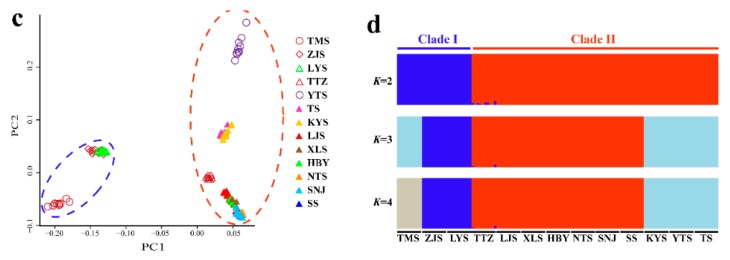
Population genetics analyses of *P. glacialis* based on genotyping-by-sequencing (GBS) data. (**a**) Sampling sites in this study representing a total of 129 individuals from 13 populations; (**b**) Neighbor joining (NJ) tree constructed using *p*-distance between individuals representing 13 populations; (**c**) Principal Components 1 and 2 for all individuals; (**d**) population genetic structure based on genomic SNPs. Each vertical bar represents one individual, and the length of each colored segment indicates the proportion of the individual genome inferred from ancestral populations (*K* = 2–4).

The NJ tree (Figure 7b) of all 129 individuals based on GBS SNP data was similar in topology to the mitochondrial gene-based Bayesian tree (Figure 3). However, Clade II of the NJ tree contained the TTZ population, which was grouped into an alternate clade (Clade B) in the mtDNA tree (Figure 3; Figure 7b). Two-way PCA also supported these groupings (Figure 7c). To further understand the population genetic structure of *P. glacialis* and determine its ancestry, we performed ADMIXTURE analysis with cross-validation errors for the number of clusters, *K*, from two to eight, and determined that *K* was optimized at a value of two (fewest cross-validation errors). Analysis at *K* = 2 revealed that populations located south of the Huaihe River were distinguished from populations in the Qinling Mountains-Huaihe River regions, and mixed SNP signals were observed in the TTZ population (Figure 7d).

## 4. Discussion

### 4.1. Phylogenetic Relationships and Divergence Times

Divergence time estimates indicated that the early divergence of *Parnassius* in the early Miocene period was likely correlated with the formation of mountain ranges (blue shades in Figure 2). During the subsequent global climate warming (mid-Miocene climatic optimum period between 17 and 15 Ma) [67], *Parnassius* subclades could have originated in refugia at higher elevations and later spread to adjacent areas with accelerated incipient speciation during cooling periods, likely associated with divergences and shifts of their host plants [68,69]. To our knowledge, *P. stubbendorfii* in China is mainly distributed in the northwest, while *P. glacialis* is distributed in the Qinling Mountains in the southeast. The distributional patterns of these two disjunct species were likely shaped by the intense orogenesis of the Qinling Mountains during the Miocene period some 10 million years ago, continuing into the Quaternary period [70]. Our estimated time tree (Figure 2) showed that the divergence of *P. glacialis* from its closest relative *P. stubbendorfii* occurred about 5.59 Ma in the late Miocene period, coincident with the rapid Qinling uplifting.

The early diversification of *P. glacialis* occurred ~0.95 Ma during the Pleistocene period, which closely coincided with the Kunlun-Huanghe tectonic movement that occurred from 1.1 to 0.6 Ma [71] and influenced the present-day pattern of the East Asian monsoon [72,73]. This geological event and associated global cooling caused widespread glacial formations in mountainous regions of China [72], triggering habitat fragmentation and restricting gene flow among *P. glacialis* populations, which are considered to be responsible for current butterfly species distribution patterns [74,75,76]. 

A positive correlation between genetic distance and geographic distance was detected in this study (Figure 4), suggesting that the genetic differentiation of *P. glacialis* is likely associated with its geographic isolation. Meanwhile, AMOVA analyses indicated little genetic variation within populations (Table 3), which probably resulted from interrupted gene flow among populations. Moreover, the higher proportion of private haplotypes observed in *P. glacialis* (82.05%, Table 1) implied the occurrence of habitat fragmentation events. 

### 4.2. Demographic History

**Figure 8:**
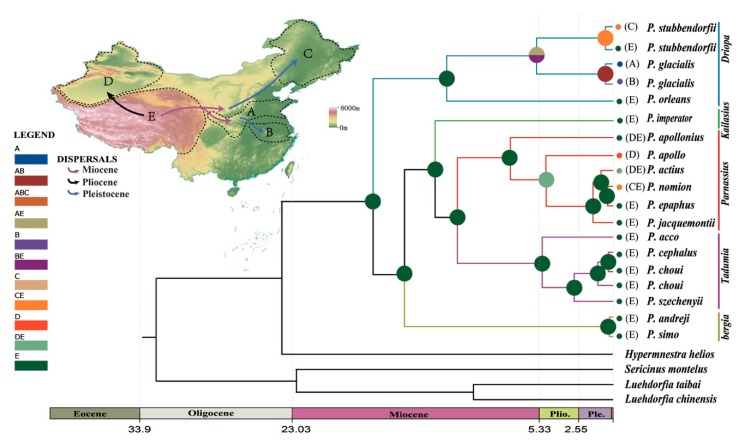
Chronogram and ancestral area reconstruction of *Parnassius* in China based on mtDNA. Ancestral area assignments at nodes represent marginal probabilities of alternative ancestral distributions obtained by statistical dispersal-vicariance analysis (S-DIVA). (**A**) Qinling Mountains-Huaihe River region; (**B**) area south of the Huaihe River; (**C**) northeast China; (**D**) Xinjiang; and (**E**) the Qinghai-Tibet Plateau (QTP). Data from C and D were not included in this report.

Ancestral area reconstruction (Figure 8) indicated that the early diversification of genus *Parnassius* occurred at the Qinghai-Tibet Plateau during the middle Miocene period. *P. glacialis* ancestors evolved from a lineage from the QTP area that spread to the Qinling Mountains-Huaihe river regions and further dispersed to areas south of the Yangtze River. The divergence within each clade of *P. glacialis* took place during middle to late Pleistocene, coinciding with repetitive glaciation events in this period. 

Clade A was mainly distributed in the Qinling Mountains and the region north of the Huaihe River. Populations from the Qinling Mountains likely dispersed eastward to colonize areas north of the Huaihe River through the Funiu Mountains during Pleistocene glacial periods. The BSP for Clade A indicated a recent expansion event that began approximately at 56 ka (Figure 7). Clade B distribution was restricted to areas south of the Huaihe River. This clade might have spread from the Qinling mountains to the present-day distributions along the Dabie mountain ranges. A unimodal mismatch distribution, significantly negative Fu’s *Fs* and Tajima’s *D,* all support the occurrence of population expansion for Clade B; demographic expansion occurred in the later Pleistocene (~44.8 ka), consistent with BSP analysis. Haplotypes from both mitochondrial clades have only been found at the junction between the Qinling and Dabie mountains (LJS) suggesting that the expansion of both clades resulted in the current sympatry observed in the LJS locality. Individuals of Clade B probably dispersed through the Dabie mountains and came into secondary contact with Clade A at LJS.

Unlike temperate species in Europe and North America that underwent expansions post Last Glacial Maximum (LGM, 25 to 15 ka), BSP analysis revealed an unusual *P. glacialis* expansion during the cooling transition between the Last Interglacial (LIG, 130 to 80 ka) and LGM, which was congruent with the results of previous studies about other insects in East Asia [77,78]. Previous paleoclimate research suggested that the glacial extent of the Marine Isotope Stage 3b (MIS 3b cold period, 54 to 44 ka) was larger than that of LGM at middle and low latitudes [79]. The larger glacial extent in MIS 3b could have facilitated long-range dispersal in this region. Populations of Clade B could have experienced a sudden demographic expansion during the subsequent interglacial period, which was likely due to the relatively lower genetic distances for this group compared to Clade A. 

### 4.3. Incongruence between Mitochondrial DNA (mtDNA) and Nuclear SNP Analysis

Mitochondrial DNA haplotype data and nuclear SNP data revealed minor phylogenetic incongruences between two populations in this study (TTZ and LJS) (Figure 1; Figure 8). Under natural conditions, the admixture of clades is known to occur in secondary contact zones, which has been well documented for spatially isolated populations in Europe and North America [4,80,81]. Population LJS displayed mixed phylogenetic signals in mtDNA haplotypes, thus, its mixed haplotypes imply the secondary contact of Clade A with Clade B after their initial divergence. The inheritance modes of nuclear and mitochondrial genes, population TTZ of Clade II (nuclear data), and corresponding Clade B (mitochondrial data) were differentially categorized, which could be attributed to the male-biased dispersal and female philopatry of *P. glacialis* [82,83]. 

## 5. Conclusions

To understand the spatiotemporal pattern of *P. glacialis* in China, we reconstruct the phylogeny, genetic structure, and demographic history of this species by using mtDNA and nuclear SNP data. The mtDNA data demonstrated that this species was estimated to have diverged about 0.95 million years ago during the middle Pleistocene transition into two main clades that likely formed during the Kunlun-Huanghe tectonic movement. The two clades then dispersed independently in distinct geographic areas alongside the mountainous routes in central and southern China, most likely driven by the Pleistocene glacial-interglacial cycles. In addition, nuclear SNP analysis was generally congruent with mtDNA results at the individual level. A minor incongruence of genetic structures was likely due to secondary contact and male-biased dispersal. Our work demonstrates that complicated dispersal-vicariance evolutionary processes likely led to the current geographic distribution of *P. glacialis* in China, particularly the uplift of the Qinghai-Tibet Plateau and related climatic oscillations during the Quaternary period.

## Figures and Tables

**Table 1:** *P. glacialis* population genetic parameters and sample data. (N, Sample size subjected to mtDNA/SNP examination; “H”, for mtDNA haplotype; Clade A/B as shown in Figure 3).

	Population	Code	N	Geographic Coordinates	Altitude (m)	Clade	Haplotypes (No. of Individuals)	Haplotype Diversity (*H_d_*)	Nucleotide Diversity (*π*)
1	Zijinshan, Jiangsu Prov.	ZJS	30/10	E118.83, N32.06	314	B	H1(19), H2(2), H3(5), H4(4)	0.568	0.00027
2	Yuntaishan, Jiangsu Prov.	YTS	30/10	E119.40, N34.71	345	A	H5(30)	0	0
3	Tiantangzhai, Anhui Prov.	TTZ	28/10	E115.77, N31.17	615	B	H6(24), H7(4)	0.254	0.00008
4	Huangbaiyuan, Shaanxi Prov.	HBY	30/10	E107.40, N33.73	1360	A	H8(2), H9(18), H10(6), H11(2), H35(1), H36(1)	0.609	0.00026
5	Laojunshan, Henan Prov.	LJS	30/10	E111.66, N33.76	861	A/B	H12(3), H13(1), H14(4), H15(14), H16(4), H17(2), H38(1), H39(1)	0.754	0.00188
6	Tianmushan, Zhejiang Prov.	TMS	26/10	E119.45, N30.34	536	B	H18(13), H19(13)	0.520	0.00052
7	Taishan, Shandong Prov.	TS	28/10	E117.12, N36.25	685	A	H20(5), H21(7), H22(16)	0.601	0.00023
8	Xiaolongshan, Gansu Prov.	XLS	29/10	E105.68, N34.85	1420	A	H9(19), H10(9), H11(1)	0.490	0.00017
9	Langyashan, Anhui Prov.	LYS	26/10	E118.29, N32.28	270	B	H2(2), H3(3), H4(5), H23(16)	0.588	0.00057
10	Niutoushan, Hubei Prov.	NTS	29/10	E110.73, N32.60	680	A	H24(5), H25(2), H13(21), H37(1)	0.456	0.00064
11	Kunyushan, Shandong Prov.	KYS	30/10	E121.73, N37.28	290	A	H26(10), H27(20)	0.460	0.00015
12	Shennongjia, Hubei Prov.	SNJ	26/10	E110.35, N31.52	1820	A	H9(1), H28(4), H29(11), H30(8), H31(2)	0.723	0.00043
13	Songshan, Henan Prov.	SS	26/9	E113.05, N34.48	716	A	H32(24), H33(1), H34(1)	0.151	0.00007

**Table 2:** Nei’s genetic distance (below diagonal) and geographic distance (above diagonal, km) between geographic populations of *P. glacialis*.

Geographic Population	ZJS	YTS	TTZ	HBY	LJS	TMS	TS	XLS	LYS	NTS	KYS	SNJ	SS
ZJS		299.38	302.38	1082.6	695.33	200.14	491.73	1257.7	56.41	761.46	638.06	803.52	601.30
YTS	0.245		514.84	1027.7	719.14	485.95	268.21	1252.2	289.14	833.26	354.38	914.00	581.63
TTZ	0.084	0.209		836.45	481.42	361.58	574.63	1026.4	264.31	502.08	868.02	518.65	446.98
HBY	0.333	0.156	0.277		393.87	1195.9	928.24	201.19	1027.7	335.31	1354.3	369.71	526.92
LJS	0.210	0.131	0.183	0.086		826.48	569.04	562.40	639.54	152.44	991.16	277.63	151.52
TMS	0.052	0.259	0.113	0.379	0.245		691.85	1382.5	242.23	862.78	799.85	877.57	757.24
TS	0.185	0.149	0.137	0.183	0.157	0.219		1046.0	454.34	709.80	426.27	816.40	417.66
XLS	0.336	0.159	0.279	0.001	0.088	0.385	0.184		1201.9	530.26	1465.8	570.81	675.03
LYS	0.035	0.288	0.138	0.398	0.274	0.070	0.247	0.399		708.36	638.48	754.12	544.95
NTS	0.326	0.137	0.267	0.055	0.068	0.335	0.196	0.057	0.399		1125.9	127.99	297.74
KYS	0.301	0.266	0.241	0.226	0.230	0.345	0.149	0.234	0.377	0.264		1223.5	840.85
SNJ	0.400	0.203	0.332	0.040	0.100	0.434	0.231	0.042	0.484	0.058	0.162		415.24
SS	0.290	0.253	0.231	0.086	0.120	0.334	0.207	0.087	0.365	0.143	0.256	0.125	

**Table 3:** Analysis of molecular variance (AMOVA) for populations of *P. glacialis* based on data from four mtDNA fragments.

Source of Variation	Variance	% Total	Fixation Indices	*p* Value
Among groups	2.44966	46.37%	*F_CT_* = 0.46373	0.0000
Among populations within groups	1.99030	37.68%	*F_SC_* = 0.70257	0.0000
Within populations	0.84257	15.95%	*F_ST_* = 0.84050	0.0016

**Table 4:** Mismatch distribution analysis and neutrality test results for Clades A and B.

	Mismatch Distribution Analysis				Neutrality Tests			τ	Expansion Time (Ma)	
	*SSD*	*P*_D_	*r*	*P*_r_	Tajima’s *D*	*P*_D_	Fu’s *Fs*	*P*_Fs_		
Clade A	0.04504	0.148	0.25367	0.316	0.43656	0.701	−22.274	0.11	3.832	-
Clade B	0.03792	0.242	0.11609	0.566	−1.39030	0.033	−27.318	0.000	3.018	0.0434

## Supplementary Materials

The following are available online at https://www.mdpi.com/2073-4425/11/2/188/s1, Table S1: Sample information for close relatives of *P. glacialis* examined in this study.
