# Supplementary material for: Spatiotemporal Differentiation of Alpine Butterfly Parnassius glacialis (Papilionidae: Parnassiinae) in China: Evidence from Mitochondrial DNA and Nuclear Single Nucleotide Polymorphisms
Source: Genes (Basel). 2020 Feb 11;11(2):188. doi: 10.3390/genes11020188 (PMC7073557; doi:10.3390/genes11020188)
Supplement: Supplementary file 1 [file genes-11-00188-s001.pdf]

genes

MDPI

Table S1. Sample information for close relatives of *P. glacialis* examined in this study.

	Species	Location	N	Geographic coordinates	Altitude (m)
1	*P. stubbendorfii*	Tonghua, Jilin Prov.	5	E125.95, N41.71	314
		Fushun, Liaoning Prov.	4	E123.91, N41.89	335
2	*P. cephalus*	Qilianshan, Qinghai Prov.	2	E98.89, N39.01	345
3	*P. imperator*	Bamishan, Gansu Prov.	8	E103.52, N35.98	615
4	*P. jacquemontii*	Qilianshan, Qinghai Prov.	2	E100.66, N38.09	1,360
5	*P. apollonius*	Jiangjunshan, Xinjiang Prov.	2	E86.09, N44.20	861
6	*P. szechenyii*	Qilianshan, Qinghai Prov.	6	E99.09, N38.34	536
7	*P. orleans*	Guanggaishan, Gansu Prov.	6	E103.21, N34.30	685
8	*P. andreji*	Qilianshan, Qinghai Prov.	7	E99.48, N38.61	1,420
9	*P. choui*	Bayankalashan, Qinghai Prov.	2	E118.29, N32.28	270
10	*P. epaphus*	Qilianshan, Qinghai Prov.	7	E100.66, N38.09	680
11	*P. acco*	Qilianshan, Qinghai Prov.	1	E99.09, N38.34	290
12	*P. apollo*	Tianshan, Xinjiang Prov.	2	E88.07, N44.11	1,820
13	*P. nomion*	Bamishan, Gansu Prov.	6	E113.05, N34.48	716
14	*P. actius*	Tianshan, Xinjiang Prov.	1	E88.07, N44.11	1,802
15	*P. simo*	Qilianshan, Qinghai Prov.	6	E100.66, N38.09	1,355
